# Use of a Mobile Phone App to Treat Depression Comorbid With Hypertension or Diabetes: A Pilot Study in Brazil and Peru

**DOI:** 10.2196/11698

**Published:** 2019-04-26

**Authors:** Paulo Menezes, Julieta Quayle, Heloísa Garcia Claro, Simone da Silva, Lena R Brandt, Francisco Diez-Canseco, J Jaime Miranda, LeShawndra N Price, David C Mohr, Ricardo Araya

**Affiliations:** 1 Population Mental Health Research Centre Department of Preventive Medicine Universidade de São Paulo São Paulo Brazil; 2 Department of Preventive Medicine University of São Paulo São Paulo Brazil; 3 Faculdade Israelita de Ciências da Saúde Albert Einstein São Paulo Brazil; 4 Centre of Excellence in Chronic Diseases Universidad Peruana Cayetano Heredia Lima Peru; 5 Department of Medicine School of Medicine Universidad Peruana Cayetano Heredia Lima Peru; 6 National Institute of Mental Health National Institutes of Health Bethesda, MD United States; 7 Center for Behavioral Intervention Technologies Northwestern University Chicago, IL United States; 8 Centre for Global Mental Health and Primary Care Research, Health Service and Population Research Institute of Psychiatry, Psychology and Neuroscience King’s College London London United Kingdom

**Keywords:** depression, mHealth, pilot study, feasibility study, PHQ-9

## Abstract

**Background:**

Depression is underdiagnosed and undertreated in primary health care. When associated with chronic physical disorders, it worsens outcomes. There is a clear gap in the treatment of depression in low- and middle-income countries (LMICs), where specialists and funds are scarce. Interventions supported by mobile health (mHealth) technologies may help to reduce this gap. Mobile phones are widely used in LMICs, offering potentially feasible and affordable alternatives for the management of depression among individuals with chronic disorders.

**Objective:**

This study aimed to explore the potential effectiveness of an mHealth intervention to help people with depressive symptoms and comorbid hypertension or diabetes and explore the feasibility of conducting large randomized controlled trials (RCTs).

**Methods:**

Emotional Control (CONEMO) is a low-intensity psychoeducational 6-week intervention delivered via mobile phones and assisted by a nurse for reducing depressive symptoms among individuals with diabetes or hypertension. CONEMO was tested in 3 pilot studies, 1 in São Paulo, Brazil, and 2 in Lima, Peru. Depressive symptoms were assessed using the Patient Health Questionnaire-9 (PHQ-9) at enrollment and at 6-week follow-up.

**Results:**

The 3 pilot studies included a total of 66 people. Most participants were females aged between 41 and 60 years. There was a reduction in depressive symptoms as measured by PHQ-9 in all pilot studies. In total, 58% (38/66) of the participants reached treatment success rate (PHQ-9 <10), with 62% (13/21) from São Paulo, 62% (13/21) from the first Lima pilot, and 50% (12/24) from the second Lima pilot study. The intervention, the app, and the support offered by the nurse and nurse assistants were well received by participants in both settings.

**Conclusions:**

The intervention was feasible in both settings. Clinical data suggested that CONEMO may help in decreasing participants’ depressive symptoms. The findings also indicated that it was possible to conduct RCTs in these settings.

## Introduction

### Background

Depression is a leading cause for the burden of disease worldwide, with 4.4% of the world’s population suffering from depression [1,2]. Depression is associated with poverty, low education, and social exclusion, all of them more common in low- and middle-income countries (LMICs) [3-5]. Depression is also associated with chronic physical diseases and disabilities [3,6]. The comorbidity of depression with other chronic diseases worsens the outcomes in physical and mental conditions, decreases quality of life, and increases the economic burden [3,7-10].

In LMICs, people with depression are commonly underdiagnosed and undertreated [5]. One in every 27 people with depression receive treatment and only 15% of these receive adequate care [11]. There are marked staff shortages in LMICs, especially of the more specialized resources such as psychiatrists, psychologists, and psychiatric nurses [5,12]. Much of this gap has to be covered through the strategy of task shifting or transferring responsibilities for the care of the mentally ill to a lower cadre of health workers [13]. However, task shifting also requires resources to identify, train, and supervise nonspecialized health care workers or lay counsellors, which are frequently not available, making it difficult to scale it up in many environments. Digital mental health technologies, which can provide treatment to patients via apps and connect patients to a care coordinator, can simplify care coordination tasks, reducing the amount of training, thereby providing a more scalable process [14]. There is a growing body of evidence that mobile interventions for common mental health problems, such as depression and anxiety, delivered through an app can be effective in reducing symptoms. Whether they are more effective when accompanied by support from a coach or care coordinator than stand-alone solutions is still controversial [15].

The Latin America Treatment and Innovation Network in Mental Health seeks to develop and test a blended intervention for depression and comorbid chronic health problems such as hypertension and/or diabetes. We developed *Emotional Control* (CONEMO), a low-intensity psychoeducational 6-week intervention delivered via mobile phones assisted by a nurse for reducing depressive symptoms among individuals with diabetes or hypertension.

The intervention aims to reduce depressive symptoms among patients with comorbid diabetes or hypertension recruited from different health service units in São Paulo (Brazil) and Lima (Peru). Therefore, the focus of this project is on providing patients tools through mobile phones, with low-intensity support that can be provided in minimal time by nurses or nurse assistants (NAs), who are available in the clinics but have no specialized training in mental health. These professionals receive specialized supervision provided by the study staff to perform their tasks. This intervention is unique in LMICs in terms of using a technological platform and integrating the care of chronic mental and physical conditions [16].

### Objectives

This pilot study aimed to:

1. Explore the potential effectiveness and feasibility of using the CONEMO intervention to help people with or hypertension or diabetes and comorbid depressive symptoms;
2. Test recruitment strategies to estimate how many participants should be screened to reach our target sample for fully powered randomized controlled trials (RCTs);
3. Assess acceptability and satisfaction of patients with the CONEMO intervention.

## Methods

### Study Design, Settings, and Participants

We conducted 3 pilot studies between 2015 and 2016, of which 1 was in São Paulo, Brazil, and 2 in Lima, Peru. São Paulo is the largest city of Brazil with 11.2 million inhabitants [17]. Brazil offers universal health coverage to its population with primary care playing a key role. The Family Health Care Strategy (FHCS) aims to provide primary care close to where inhabitants live through family health teams composed of 1 family or general physician, 1 nurse, 2 NAs, and 5 to 6 community health agents [18]. Currently, there are more than 43,000 Family Health Teams covering 65% of the total population in the country and 40% of the population in the city of São Paulo [19]. A large survey estimated that approximately 10% of the population in São Paulo fulfilled criteria for major depression in the past 12 months, with 80% presenting moderate-to-severe depression [20]. A recent census indicated that Brazil currently has 5 psychiatrists per 100,000 inhabitants, but the majority work only in the private sector.

Lima, the capital of Peru, has approximately 9 million inhabitants, one-third of the total Peruvian population [21]. The prevalence of depression has been estimated as 17.5% [22]. In Peru, health care is offered both in public and private sectors, from the primary to tertiary care level, and although the current mental health reform is aiming at shifting mental health care toward a community approach, the majority of mental health care is still restricted to tertiary care–level facilities [23]. The Peruvian health care system is administered by different entities: the Ministry of Health (MINSA; covering 60% of the population), EsSalud, which is a social and health insurance for employees (30% coverage), and armed forces, police, and private sector (covering the remaining 10%) [24]. In Peru, there are 0.6 psychiatrists per 100,000 inhabitants, well below the average for LMICs.

In São Paulo we conducted the pilot study with 4 teams from 2 FHCS clinics. In Lima, the first pilot study took place in the endocrinology and cardiology outpatient consultation area of a tertiary-level hospital from the MINSA, whereas in the second pilot, patients were recruited in 2 primary care health centers, mostly in the elderly adult consultation programs, where people with noncommunicable diseases are monitored, at EsSalud.

Participants were eligible according to the following inclusion criteria:

1. Aged 21 years or above;
2. Had hypertension or diabetes;
3. Had depressive symptoms, evaluated by the PHQ-9 (score ≥10); and
4. Self-reported reading ability in Portuguese (São Paulo) or Spanish (Lima).

The exclusion criteria were as follows:

1. Psychosis symptoms detected by a 5-item screening questionnaire;
2. Pregnancy (self-reported);
3. High suicidal risk detected by a positive answer in item 9 of the PHQ-9 followed by a protocol to assess suicidal risk (Suicide Risk Assessment Protocol); and
4. Cognitive impairment detected by a 4-item questionnaire.

We used convenience samples in the 3 pilot studies. We envisaged that with 20 participants in each pilot, we would be able to achieve the aims proposed.

### Measurements

We collected information about socioeconomic and demographic characteristics, depressive symptoms, suicidal ideation, presence of psychosis symptoms, cognitive impairment, quality of life, adherence to medical treatment, and disabilities (Table 1).

To explore the potential effectiveness of the CONEMO intervention, we used the Patient Health Questionnaire-9 (PHQ-9) as the main measure of outcome [25]. We applied the PHQ-9 at baseline and at the end of the 6-week intervention to assess severity of depressive symptoms.

To rule out the presence of psychotic symptoms, we used the Psychosis Screening Questionnaire [26]; to assess cognitive impairment, we used the Community Screening for Dementia [27]; and to estimate quality of life, the EuroQoL (Quality of Life)-5 dimensions (EQ-5D) was used [28]. Disabilities were assessed with the World Health Organization Disability Assessment Schedule II [29].

**Table 1:** Description of instruments used in the pilot studies, time of application, and purpose (São Paulo, Brazil, and Lima, Peru).

Instrument	Time	Purpose
First informed consent (S1^a^)	Prescreening	Introduction of the study and obtaining consent for screening
PHQ^b^-2/PHQ-9/S-RAP^c^ (S2^d^)	Screening	Assessment of depressive symptoms and suicidal ideation
Second informed consent (B1^e^)	Before baseline	Consent to the study
PHQ-9	Screening/risk assessment (1 and 3 weeks)/follow-up	Full screening for depressive symptoms
S-RAP	Screening/risk assessment (1 and 3 weeks)/follow-up	Assess Suicidal Risk
PSQ^f^	Screening	Assess the presence of psychosis
CSI-D^g^	Screening	Evaluate cognitive impairment
Demographic and socioeconomic characteristics	Baseline	Information about participants’ socioeconomic and family background
Clinical data	Baseline	Medical history
WHODAS^h^ II	Baseline/follow-ups	Assess functional disability
EQ-5D^i^	Baseline/follow-ups	Assess quality of life

^a^S1: prescreening

^b^PHQ: Patient Health Questionnaire.

^c^S-RAP: Suicide Risk Assessment Protocol.

^d^S2: screening.

^e^B1: baseline.

^f^PSQ: Psychosis Screening Questionnaire.

^g^CSI-D: Community Screening for Dementia.

^h^WHODAS: World Health Organization Disability Assessment Schedule.

^i^EQ-5D: EuroQoL (Quality of Life)-5 dimensions.

We also used data from participants captured automatically by the CONEMO system to monitor patients’ progress, including completion of sessions, intervals between accessing sessions, or sessions missed. We collected information from participants about the acceptability and satisfaction with the CONEMO app and the dashboard through a questionnaire using a Likert scale of 5 points, where 1 is *disagree* and 5 is *agree*, at the end of the pilot study.

### Emotional Control (CONEMO) Intervention

**Figure 1:**
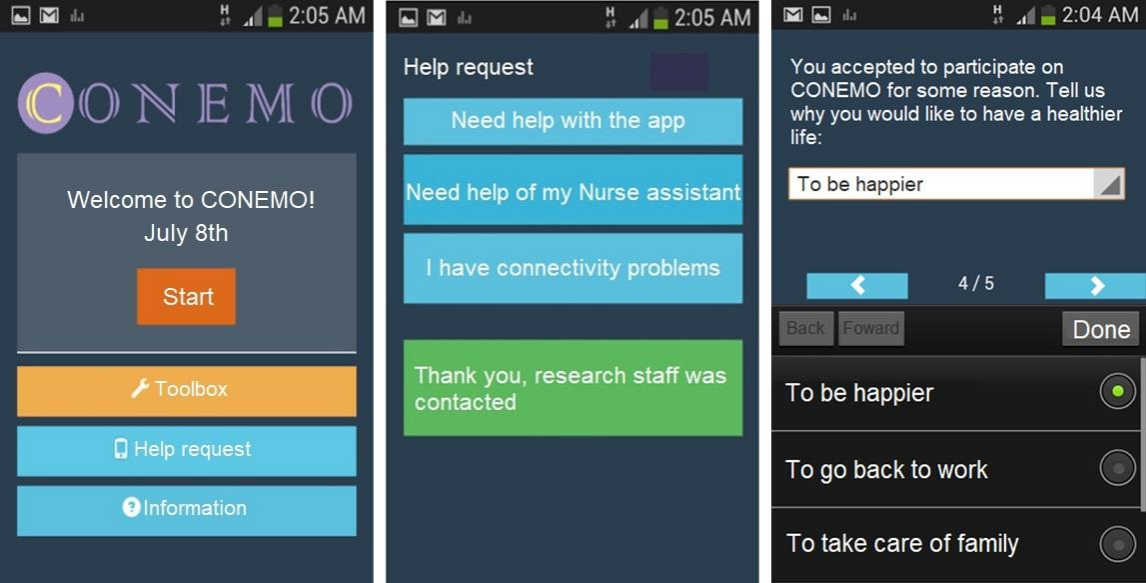
CONEMO app screenshots. CONEMO: Emotional Control.

We tested an intervention that could be delivered in existing general medical care settings by available local personnel. The choice of a psychological intervention as the main component relates to the public health need for these approaches in most LMICs. CONEMO is a mobile phone-delivered intervention, supported by an NA or a nurse, using a task-shifting model, as recommended by the World Health Organization [13,30]. CONEMO consists of 18 behavioral activation sessions, delivered over 6 weeks (3 sessions per week). As part of the behavioral activation program, CONEMO aims at increasing daily life activities, especially pleasant ones, healthy ones, and tasks, as well as providing further information and health self-care messages, and increasing motivation (Figure 1). Participants received a device from the project, with the CONEMO app installed, for use during the 6-week program, which had to be returned to the respective nurse after that period.

The tasks of the nurse or NA were as follows: (1) an initial contact by phone to schedule a training session for the participant; (2) a face-to-face training session to introduce the CONEMO app; (3) a monitoring call after week 1 to answer possible queries from participants and to reinforce motivation to participate; (4) a monitoring call after week 4 with the same objectives as above; (5) a phone call to schedule the closing meeting; and (6) a final face-to-face appointment with the participant to finish the intervention and collect the mobile phone. Nurses or NAs received weekly face-to-face supervision from research clinical psychologists.

### Data Analysis

To test recruitment strategies, we analyzed the data gathered during the process of recruiting and enrolling participants (Aim 2). The comparison of participants’ PHQ-9 scores at baseline and at follow-up allowed a preliminary assessment of potential effectiveness (Aim 1), and the number of sessions completed and ratings on the satisfaction with the CONEMO intervention gave us an insight into the acceptance of the CONEMO intervention (Aim 3).

The main outcome measure was patients’ PHQ-9 scores at the end of the 6-week intervention. We considered that patients had recovered when the PHQ-9 score at follow-up was <10. The secondary outcomes, such as disability levels and quality of life, were also examined at the end of the pilots. Descriptive statistics were also used to assess participants’ perceptions at the end of the study. We conducted all data analysis using STATA 11 by StataCorp [31].

### Ethical Considerations

The pilot studies were approved by local Institutional Review Boards and the US National Institute of Mental Health Data and Safety Monitoring Board. Participants consented to participate in the study and interviews took place after collecting the participants’ signatures on informed consent forms. All investigators and research assistants involved in the data collection and analysis completed ethics and human research good clinical practices training before starting their activities in the project.

## Results

### Assessment of Recruitment Strategy

**Figure 2:**
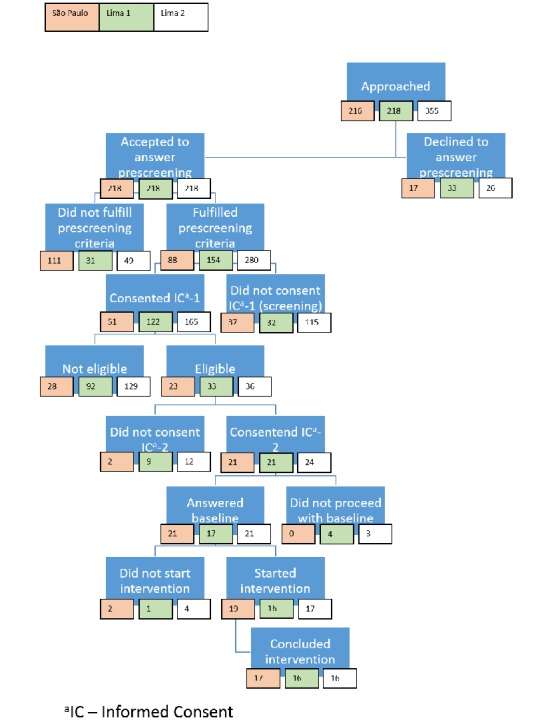
Flow chart of recruitment and intervention in Lima, Peru, and São Paulo, Brazil. IC: informed consent.

We carried out 1 pilot study in São Paulo and 2 pilot studies in Lima. In São Paulo, 4 nurses from the 2 participating FHCS clinics were responsible to support the CONEMO intervention for participants recruited into the study. In the first pilot in Lima, we employed a nurse to support the delivery of the intervention, whereas in the second pilot study in Lima, we worked with 6 nurses employed by 2 public health care facilities. We included 66 patients in the 3 pilot studies after approaching 793 people (Figure 2). Of the 216 subjects approached in São Paulo, 9.7% (21/216) were included in the pilot study. In the 2 pilot studies in Lima, we approached 577 subjects and included 7.7% (45/577), 21 in the first and 24 in the second pilot studies. To recruit participants, we spent 30 days in the field in São Paulo, 21 days in Lima’s first pilot study, and 24 days in Lima’s second pilot study. Most participants were women aged between 41 and 60 years (Table 2). In São Paulo, participants had a lower educational level than those in Lima.

**Table 2:** Socioeconomic and demographic characteristics of participants included in the pilot studies in São Paulo, Brazil, and Lima, Peru.

Characteristics		São Paulo (N=21), n (%)	Lima—pilot 1 (N=21), n (%)	Lima—pilot 2 (N=24), n (%)	Total (N=66), n (%)
**Age (years)**					
	21-40	3 (14)	0 (0)	1 (4)	4 (6)
	41-60	11 (52)	13 (62)	11 (46)	35 (53)
	≥61	7 (33)	8 (38)	12 (50)	27 (41)
**Gender**					
	Male	5 (24)	8 (38)	6 (25)	19 (29)
	Female	16 (76)	13 (62)	18 (75)	47 (71)
**Chronic disease**					
	HBP^a^	14 (67)	1 (5)	9 (38)	24 (36)
	DM^b^	1 (5)	12 (57)	6 (25)	19 (29)
	HBP and DM	6 (28.6)	8 (38)	9 (38)	23 (35)
**Educational level**					
	Primary school	12 (57)	3 (14)	4 (17)	19 (29)
	High school	6 (29)	8 (38)	5 (21)	19 (29)
	Technical course	3 (14)	4 (19)	7 (29)	14 (21)
	University or postgraduate	0 (0)	2 (10)	5 (21)	7 (11)
	Unknown	0 (0)	4 (19)	3 (13)	7 (11)
**Income (Brazilian minimum wage)**					
	0-4	14 (67)	—^c^	—	14 (21)
	>4	7 (33)	—	—	7 (11)
**Income Peru (Soles)**					
	0-1500	—	9 (42.9)	7 (29)	16 (24)
	>1500	—	7 (33.3)	12 (50)	19 (29)
	Do not know/did not answer	—	5 (23.8)	5 (21)	10 (15)

^a^HBP: high blood pressure (hypertension).

^b^DM: diabetes mellitus.

^c^—: not applicable.

### Potential Effectiveness and Feasibility of Emotional Control (CONEMO) Intervention

**Figure 3:**
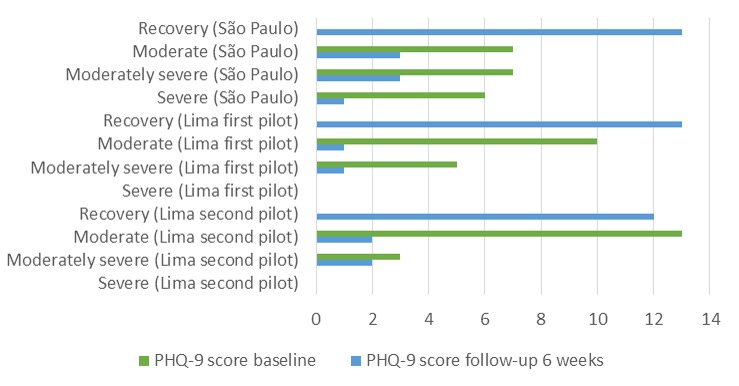
Severity of depressive symptoms at baseline and 6-week follow-up, according to Patient Health Questionnaire-9 (PHQ-9) scores, for the 3 pilot studies (São Paulo, Brazil, and Lima, Peru).

At the 6-week follow up, there was 5% (1/21%) loss to follow-up in São Paulo and 28.6% (6/21) in the first and 33% (8/24) in the second Lima pilot studies. Among participants who completed follow-up assessments, 11 out of 20 (55%) had moderately severe or severe symptoms of depression at baseline in São Paulo (Figure 3). In Lima, considering both pilots, none had severe symptoms at baseline and 8 out of 31 (26%) had moderately severe symptoms. At follow-up assessment, 65% (13/20) of participants had recovered from depression (PHQ-9 <10) in São Paulo, 87% (13/15) in the first Lima pilot study, and 75% (12/16) in the second Lima pilot study (Figure 3). Only 2 out of 16 (13%) participants in Lima’s second pilot study worsened their level of depressive symptoms. Furthermore, 6 out of 20 participants (30%) presented mild suicide risk at baseline in São Paulo, whereas in Lima none of the participants presented suicide risk. In the follow-up assessments of the 3 pilot studies, no participant presented suicide risk.

In São Paulo and Lima, all participants presented some level of functional disability at baseline, with more than 50% presenting moderate or severe functional disability. In all pilot studies, there was a trend toward a reduction in levels of functional disability at the follow-up assessments (Table 3). Regarding quality of life, we observed small inconsistent variations on EQ-5D scores from baseline to 6-week follow-up (Table 3).

**Table 3:** Functional disability and quality of life during baseline and at 6-week follow-up in São Paulo, Brazil, and Lima, Peru.

Scales and categories		São Paulo (N=20), n (%)		Lima—pilot 1 (N=15), n (%)		Lima—pilot 2 (N=16), n (%)	
		Baseline	6-week follow-up	Baseline	6-week follow-up	Baseline	6-week follow-up
**World Health Organization Disability Assessment Schedule**							
	No disability	0 (0)	2 (10)	0 (0)	1 (7)	0 (0)	0 (0)
	Mild disability	9 (45)	10 (50)	5 (33)	9 (60)	7 (44)	9 (56)
	Moderate disability	8 (40)	5 (25)	7 (47)	5 (33)	7 (44)	7 (44)
	Severe disability	3 (15)	3 (15)	2 (13)	0 (0)	2 (13)	0 (0)
**EuroQoL (Quality of Life)-5 dimensions**							
	Problems to walk	10 (50)	11 (55)	10 (67)	7 (47)	7 (44)	5 (31)
	Problems washing or dressing	9 (45)	5 (25)	3 (20)	2 (13)	1 (6)	0 (0)
	Problems with performing usual activities	9 (45)	9 (45)	8 (53)	4 (27)	5 (31)	5 (31)
	Moderate or extreme pain/discomfort	14 (70)	16 (80)	14 (93)	11 (73)	13 (81)	11 (69)
	Moderate or extreme anxiety/depression	17 (85)	15 (75)	7 (47)	7 (47)	12 (75)	8 (50)

**Figure 4:**
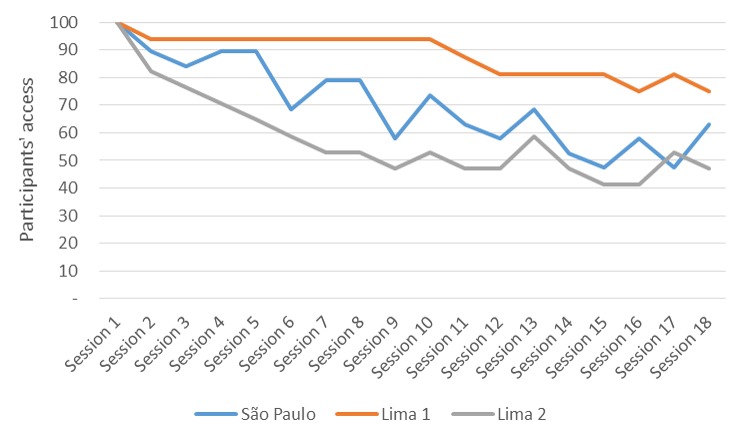
Percentage of participants who accessed the CONEMO app sessions in the pilot studies in São Paulo, Brazil, and Lima, Peru. CONEMO: Emotional Control.

Access of participants to the CONEMO sessions decreased progressively over time in the São Paulo and in the second Lima pilot study (Figure 4), whereas in the first Lima pilot study, access started to decrease only after 4 weeks of the intervention. Percentages of completion of sessions were 70% in São Paulo and 88% and 58% in the first and second pilot studies in Lima, respectively. In São Paulo, 87% of the participants accessed all first 6 sessions, whereas 95% and 76% did so in the first and second pilot studies in Lima, respectively. Furthermore, 69% of participants in São Paulo and 91% and 50% in Lima’s first and second pilot studies, respectively, accessed sessions 7 to 12. Adherence decreased for sessions 13 to 18, with 55% in São Paulo and 80% and 48% in the first and second pilot studies in Lima, respectively.

The CONEMO app (participant interface) worked adequately during the trial, despite some connectivity issues when receiving information on participants’ performance. These issues did not affect the intervention or data collection.

We collected data on acceptability and satisfaction with CONEMO from 20 participants in São Paulo, 15 in Lima’s first pilot, and 16 in Lima’s second pilot. The evaluation of the intervention by participants was generally positive: all mean ratings were above 3.5 points (maximum of 5 points), both in São Paulo and Lima. Patients considered that the objectives were attained and that the intervention helped their physical and mental health and helped them to get organized. In their opinion the duration of the intervention, the amount of contacts with the nurse or NA, and the training received were adequate (Table 4).

Participants considered the CONEMO app easy to use, were able to access videos and other resources included in the app, and thought the size of fonts and layout of the app was appropriate (Table 5). They also assessed the role of nurses or NAs in the task-shifting strategy proposed in the CONEMO intervention. They considered training, procedures, and availability of nurses or NAs as sufficient or good. Around 90% of the participants in all pilots considered it important to have nurses or NAs’ support.

**Table 4:** Evaluation of participants regarding CONEMO (Emotional Control) intervention in the pilot study in São Paulo, Brazil, and Lima, Peru.

Intervention aspects evaluated	São Paulo (average grade)	Lima pilot study 1 (average grade)	Lima pilot study 2 (average grade)
Helped with physical health	4.1	4.0	3.8
Helped with mental health	4.4	4.4	4.3
Helped having the will to do things	3.9	4.3	4.3
Helped to get organized	3.6	4.3	4.3
Count on nurse assistant or nurse	4.5	4.1	4.1
Nurse assistant or nurse helped	4.4	4.3	3.8
Number of contacts	4.1	3.6	3.2
Training quality	4.1	3.7	3.1
Intervention duration	4.1	2.8	2.9
Achievement of objectives	4.1	4.2	4.2
Satisfaction with results	4.3	4.4	4.3
Would indicate to a friend	3.6	4.6	4.5

**Table 5:** Evaluation of participants regarding technological aspects of the CONEMO (Emotional Control) app used during the pilot studies in São Paulo, Brazil, and Lima, Peru.

Technological aspect	São Paulo (average grade)	Lima pilot study 1 (average grade)	Lima pilot study 2 (average grade)
Easy to use	4.4	4.3	3.8
Usefulness	4.6	4.7	4.4
Adequacy of frequency of sessions	4.3	3.3	3.9
Content interesting	4.4	4.6	4.5
Video quality	4.4	4.6	4.4
Sound quality	3.9	4.6	4.3
Choices presented	4.1	4.6	4.5
Training session	4.3	4.0	4.3
Reminders	3.6	3.0	4.3

## Discussion

### Principal Findings

The main aim of this pilot study was to explore the feasibility and potential effectiveness of the CONEMO intervention for depressed people with comorbid hypertension or diabetes. We also wanted to assess the feasibility of undertaking fully powered RCTs to test the effectiveness of this intervention. We found that patients were able to use the CONEMO app, that they tended to show improvement in the severity of depressive symptoms by the end of the intervention, and that they were satisfied with the intervention. Our results are encouraging and suggest that the intervention is feasible and potentially effective with this population.

To carry out RCTs, we needed to ensure that we would be able to recruit participants in the numbers needed. On average, out of every 13 participants screened, we were able to include 1 eligible participant in the study. In São Paulo and Lima, recruitment took place in primary health care units and, additionally, in the outpatient consultation areas of a public hospital in the first pilot study in Lima, with patients presenting different conditions apart from hypertension and diabetes. In both sites, many participants were unable to read or write. As a result, it was necessary to prescreen twice as many patients than we first anticipated to reach the numbers we had planned for each pilot study. This information allowed us to prepare a more realistic recruitment strategy and plan.

The instruments and measures were well understood by participants, and research assistants also provided positive feedback on their use. The outcome measures seemed sensitive to change and allowed a useful characterization of the study sample. The research assistants did not refer problems from the participants in understanding the instructions or questions included in the study. As only 1 participant showed cognitive impairment or psychotic symptoms in the screening, the assessment of these conditions may not be needed in the full trials. This will decrease the time taken for screening interviews, also reducing costs for running the trials.

Overall, there was a noticeable trend in all pilot studies for a reduction on depressive symptoms over time, as measured by the PHQ-9. Our approach was categorical, using PHQ-9 cutoff points to decide on caseness and severity. The proposed measure of clinical success, having a PHQ-9 score ≤9 at follow-up (recovery), was considered a stringent but good measure. Nevertheless, all our estimates need to be taken with caution as this pilot study was not powered to detect any statistically significant changes.

Other secondary measures of success of the intervention also yielded some results in the expected direction. Disability levels seemed to improve by the end of the intervention. This trend was not as clear for the quality of life measure (EQ-5D).

This study also found that participants were satisfied with the intervention and nurse or NA support. Other studies have also shown benefits in the use of collaborative care and task-shifted approaches in the treatment of depression [32-34]. Participants found the CONEMO app useful, easy to use, and with interesting content. There was a decrease in adherence to the sessions over time, which is consistent with a large majority of digital mental health interventions. This suggests a need to review the content and organization of the sessions before the start of the RCTs. Changes should include shortening sessions, making language simpler, and adding more video material. Reviewing our strategy for persuasive design could also improve longer term adherence, such as including more tailoring and personalization, improving the quality of automated notifications (eg, positive reinforcement for engagement), and leveraging contact with the nurse coordinator. We are hopeful that these changes will improve session adherence in the RCTs.

### Strengths and Limitations

To the best of our knowledge, this is the first study that pilot tested a mobile intervention for symptoms of depression among individuals with chronic conditions in 2 settings of middle-income countries of Latin America. Our samples were small, so the pilot studies were not powered to test the efficacy of the intervention; however, our results were in the predicted direction, and the proportions of treatment success in each sample are useful to calculate full trial sample sizes. We found that many potentially eligible participants could not participate because of difficulties to read or write. This could potentially affect the generalizability of the results. However, with the increasing literacy trend in younger generations in Latin America, these difficulties will decrease, and more people will be able to benefit from similar apps in the future.

### Conclusions

The technological system, CONEMO, seems feasible to use in these settings with different languages to help patients with diabetes or hypertension and comorbid depressive symptoms. The results from the 3 pilot studies are promising and support the implementation of fully powered trials. The CONEMO intervention will be one of the first evidence-based mobile interventions tested in large samples in 2 different settings in LMICs.

## Abbreviations

CONEMO: Emotional Control
EQ-5D: EuroQoL (Quality of Life)-5 dimensions
FHCS: Family Health Care Strategy
LMIC: low- and middle-income country
mHealth: mobile health
MINSA: Ministry of Health
NA: nurse assistant
PHQ-9: Patient Health Questionnaire-9
RCT: randomized controlled trial
